# The retention of health human resources in primary healthcare centers in Lebanon: a national survey

**DOI:** 10.1186/1472-6963-12-419

**Published:** 2012-11-22

**Authors:** Mohamad Alameddine, Shadi Saleh, Fadi El-Jardali, Hani Dimassi, Yara Mourad

**Affiliations:** 1 Assistant Professor, Faculty of Health Sciences, Department of Health Management and Policy, American University of Beirut, Riad El-Solh, Beirut, 1107 2020, Lebanon; 2 Associate Professor, Faculty of Health Sciences, Department of Health Management and Policy, American University of Beirut, Riad El-Solh, Beirut, 1107 2020, Lebanon; 3 Assistant Professor, School of Pharmacy, Lebanese American University, P.O.Box 36, Byblos, Lebanon; 4 Research Assistant, Faculty of Health Sciences, Department of Health Management and Policy, American University of Beirut, Riad El-Solh, Beirut, 1107 2020, Lebanon

**Keywords:** Retention, Human resources, Primary healthcare, Professional burnout, Lebanon

## Abstract

**Background:**

Critical shortages of health human resources (HHR), associated with high turnover rates, have been a concern in many countries around the globe. Of particular interest is the effect of such a trend on the primary healthcare (PHC) sector; considered a cornerstone in any effective healthcare system. This study is a rare attempt to investigate PHC HHR work characteristics, level of burnout and likelihood to quit as well as the factors significantly associated with staff retention at PHC centers in Lebanon.

**Methods:**

A cross-sectional design was utilized to survey all health providers at 81 PHC centers dispersed in all districts of Lebanon. The questionnaire consisted of four sections: socio-demographic/ professional background, organizational/institutional characteristics, likelihood to quit and level of professional burnout (using the Maslach-Burnout Inventory). A total of 755 providers completed the questionnaire (60.5% response rate). Bivariate analyses and multinomial logistic regression were used to determine factors associated with likelihood to quit.

**Results:**

Two out of five respondents indicated likelihood to quit their jobs within the next 1–3 years and an additional 13.4% were not sure about quitting. The top three reasons behind likelihood to quit were poor salary (54.4%), better job opportunities outside the country (35.1%) and lack of professional development (33.7%). A U-shaped relationship was observed between age and likelihood to quit. Regression analysis revealed that high levels of burnout, lower level of education and low tenure were all associated with increased likelihood to quit.

**Conclusions:**

The study findings reflect an unstable workforce and are not conducive to supporting an expanded role for PHC in the Lebanese healthcare system. While strategies aiming at improving staff retention would be important to develop and implement for all PHC HHR; targeted retention initiatives should focus on the young-new recruits and allied health professionals. Particular attention should be dedicated to enhancing providers’ role satisfaction and sense of job security. Such initiatives are of pivotal importance to stabilize the workforce and ensure its longevity.

## Introduction

Thirty years after the Alma-Ata Declaration, which officially launched Primary Healthcare (PHC) in 1978, PHC remains high on the international agenda. It was the theme of the World Health Report in 2008 entitled “Primary Health Care: now more than ever” [1]. This renewed commitment to PHC stems from the universal belief that PHC is the most effective and efficient approach to maintain population health and prevent disease progression [2-4]. Yet, the success of these renewed attempts to rejuvenate PHC services is dependent on adequate and well-qualified health human resources (HHR) and the presence of evidence-based retention strategies [5]. Although the role of PHC has lately been expanding in many countries, human resource management has not been awarded the same well-merited attention [6].

This study surveys PHC HHR to explore factors significantly associated with their retention in PHC centers in Lebanon, a subject not yet systematically investigated.

## Background

### The global shortage of health human resources

Being the crucial core of any health system, the presence of an adequate number of well trained and properly experienced HHR is central to the delivery of patient-centered healthcare services across the continuum of healthcare [5,7]. HHR literature concurs in reporting critical shortages of health workers in many countries around the globe, particularly shortages in physicians and nurses [8-10]. Such shortages are confirmed within the context of the Eastern Mediterranean Region (EMR), particularly in low and middle income countries [11].

Most medical interventions require the services of doctors, nurses and other health professionals. Their performance is the first determinant of the quality, efficacy, efficiency, accessibility and viability of health services [2,7].Studies that examined the hospital context established a link between HHR densities and health outcomes; including quality of care, morbidity and mortality [7,12]. Furthermore, HHR account for a high proportion of budgets assigned to the health sector, and health expenditures are strongly linked to the ways in which HHR are deployed and used. Finally, the competences and availability of an appropriate workforce is a critical prerequisite for the growth and development of any organization [5]. Still, HHR has been largely a neglected component of the health-system development; in best cases not given the due importance that they deserve.

### The retention of the primary healthcare workforce

Literature over the past two decades has examined the escalating PHC HHR crisis, elaborating on the centrality of recruitment and retention of PHC HHR for building balanced, effective and sustainable primary care services [9,13-15]. Moreover, the role of HHR has been acknowledged to be even more important in community-based care, which tends to use less advanced equipment and is more dependent on competent personnel [5].

The most well documented cause of turnover among health professionals is related to job dissatisfaction [16,17], to which various aspects are attributed. Literature presents a plethora of such factors that influence the retention of HHR; they could be summarized under the following three main categories: organizational characteristics, work characteristics, and individual characteristics. Organizational factors documented include salaries and benefits [18,19], along with organizational commitment and managerial support [20,21]. Work characteristics revolved around the nature of the job, which includes the workload, work environment, and work group cohesion [17,20] as well as opportunities for professional development [18,19,22]. Individual socio-demographic characteristics associated with turnover included age, education, professional position, and tenure [18,20,23].

### The primary healthcare context in Lebanon

The first call to the building of a Lebanese PHC system goes back to 1972 [24]. Since then, significant progress has been witnessed on both the public and private fronts as evident by the proliferation and expansion of PHC facilities across the Lebanese territories. On the public front, the Lebanese Ministry of Public Health led the establishment of a national PHC network that includes 140 centers [25]. The Ministry also led multiple initiatives to strengthen the PHC system, including: partnership for delivery with municipalities and private centers, the development of guidelines and health education materials, distribution of vaccines, drugs, supplies and equipment, as well as training activities [25].

Furthermore, the last few years witnessed the strengthening of privately owned and operated networks of PHC center. This astonishingly fast growth made private PHC centers the major provider of PHC services in Lebanon and has motivated the public sector to contract with a number of private centers for the provision of publicly funded PHC.

Although increased interest in the PHC sector in Lebanon has instigated the writing of a number of research reports over the last two decades [26,27], none have systematically examined factors significantly associated with retention in this vital sector.

This study aims at investigating PHC health providers’ work characteristics, level of burnout and likelihood to quit and identify the factors that are significantly associated with staff retention at PHC centers in Lebanon. To our knowledge, this is the first study to address this subject in Lebanon and the EMR.

## Methods

### Research design

A cross-sectional design was utilized to survey all health providers in 81 PHC centers dispersed in all districts and governorates of Lebanon.

### The setting and participants

Six major private networks providing primary healthcare services in Lebanon were identified and invited to participate in this study. Four agreed to participate; the other two did not for administrative and logistic purposes. The four participating networks have a large beneficiary base, running a total of 81 PHC centers dispersed nationally in all 26 districts of Lebanon. These networks are estimated to employ close to half of the primary healthcare providers working in the private sector in Lebanon.

These PHC centers offer health maintenance, promotion and prevention services, as well as a wide range of curative health services including access to generalists, specialists, diagnostic and dental services in various Lebanese areas with service coverage in both rural and urban settings. Although the health promotion and disease prevention services have been expanding over the last few years, it is noteworthy to mention that the focus of PHC centers services in Lebanon has been on delivery of curative and diagnostic services. Furthermore, active public participation remains deficient and indeed needs to be strengthened in the near future.

The target population within these centers was all health providers including: general practitioners, medical specialists (cardiologists, gynecologists, pediatricians, general surgeons and others), nurses, dentists, laboratory and radiation technologists, nutritionists and providers from others specialties (dental assistants, midwives, etc.). In each PHC center, all providers with a minimum of six months of cumulative work experience in the PHC setting were invited to fill the questionnaire. Non-providers, those involved in the management and administration of the PHC centers, were excluded.

Public PHC centers were excluded from this study for several reasons, mainly due to private PHC centers providing the majority of PHC services in Lebanon [26] and the minimal variability in recruitment and retention policies across public PHC centers. Indeed, there has been a freeze on HHR recruitment and very little turnover with respect to current staff at public PHC centers, whereby all staff are salaried governmental employees. In contrast, evidence shows variability across private PHC centers with respect to recruitment and retention policies.

### Ethical approval

The study protocol, data collection instruments and consent forms were reviewed and received approval by the Social and Behavioral Sciences Institutional Review Board at the American University of Beirut. Written consent was obtained from all respondents, by ticking on the ‘I agree’ box on the cover page of the questionnaire.

### The survey instrument

Data was collected through a questionnaire consisting of four sections: (1) socio-demographic and professional background, (2) organizational and institutional characteristics, (3) likelihood to quit, and (4) level of burnout using the Maslach Burnout Inventory (MBI). The questionnaire was reviewed and approved for content validity by a multi-disciplinary expert panel consisting of a health management and policy researcher, a clinician, nurses and a statistician. The questionnaire was translated to Arabic then back translated to English and compared to the original. No major differences were found. All language versions of the questionnaire were pilot-tested on 40 PHC providers (excluded from the study) to check for clarity of questions and to ensure that all aspects intended to be measured were covered. No major changes were made.

The dependent variable in this study, likelihood to quit, was measured by asking surveyed PHC providers to answer the following question “How likely are you to quit your current job in the next 1–3 years”. Responses were measured on a five-point Likert scale with the following options: Very unlikely, unlikely, not sure, likely, very likely. This measure of likelihood/intention to quit is well documented in literature [21,28-31].

### The maslach burnout inventory

The MBI is the most frequently used instrument to measure occupational burnout. It is a 22-item self-assessment tool that measures burnout syndrome across three subscales: emotional exhaustion-EE (9 questions), depersonalization-DP (5 questions) and personal accomplishment-PA (8 questions). The EE subscale measures workers’ feelings of emotional weariness from one’s work. The DP subscale measures workers’ impersonal manner in responding to recipients of services. The subscale of PA measures workers’ feelings of professional achievement and experience of intrinsic values at work.

The questions were answered according to a seven-point frequency scale ranging from “Never” (given a score of zero) to “Daily” (given a score of six). The scores for the questions related to each of the three subscales were added to compute a subscale score, which was then compared to the provided cut-off points for medical professions to establish the corresponding level of burnout [32].

### Data collection

The principal investigators met with the general administration of the four PHC networks in order to explain the objectives of the study. Upon obtaining approval of participation, managers at the selected PHC centers were asked to distribute the questionnaires to all providers fitting the inclusion criteria. Completion of the questionnaire was voluntary. Anonymity of respondents was maintained, with no personal identifiers used and questionnaires returned in sealed envelopes. A total of 755 out of 1247 PHC providers completed the questionnaire (response rate 60.5%).

### Data analysis

Upon completion of data collection, a random sample of 10% of the questionnaires was checked for completeness and accuracy to guarantee the quality and integrity of the collected data. Full data was then exported to and analyzed using the Statistical Package for the Social Sciences (SPSS), version 19.0. Sample characteristics were summarized using frequency and percentages. Likelihood to quit was the outcome variable of interest, and a multinomial logistic regression model was used to explain factors associated with the likelihood to quit. All analyses were conducted at a 0.05 significance level.

## Results

### Sample description

The demographic, socio-economic and professional characteristics of the respondents are shown in Table 1. The gender distribution of the sample was almost half male (49.6%) and half female (50.3%). The majority of the respondents (29.9%) fell in the relatively young age group of 26 to 35 years of age, and nearly two thirds (65.0%) were married.

**Table 1:** Demographic and professional characteristics of survey respondents (n=755)

**Variable**	**N (%)**
**Gender**	
Male	374 (49.6%)
Female	380 (50.3%)
Missing	1 (0.1%)
**Age group**	
18-25	84 (11.1%)
26-35	226 (29.9%)
36-45	207 (27.4%)
46-55	185 (24.5%)
> 55	45 (6.0%)
Missing	8 (1.1%)
**Marital status**	
Single	223 (29.5%)
Married	491 (65.0%)
Others	39 (5.2%)
Missing	2 (0.3%)
**Education**	
Intermediate/High School	15 (2.0%)
Vocational or diploma	95 (12.7%)
Nursing Degree	209 (27.9%)
University Degree	109 (14.5%)
Medical Degree	135 (18.0%)
Medical Specialty	187 (24.9%)
**Current position in PHC**	
Generalist (includes dentists)	174 (23.0%)
Medical Specialist	164 (21.7%)
Nurse	247 (32.7%)
Allied Health Professional	114 (15.1%)
Other health professional	56 (7.4%)
**Number of years spent working in PHC**	
Six months to 1 year	93 (12.3%)
1 to 5 years	271 (35.9%)
6 to 10 years	170 (22.5%)
More than 10 years	212 (28.1%)
Missing	9 (1.2%)
**Number of years working in current PHC center**	
Six months to 1 year	136 (18.0%)
1 to 5 years	331 (43.8%)
6 to 10 years	145 (19.2%)
More than 10 years	121 (16.1%)
Missing	22 (2.9%)
**Employment status at PHC**	
Permanent- Full time	428 (56.7%)
Permanent- Part time	256 (33.9%)
Temporary/Casual	62 (8.2%)
Missing	9 (1.2%)
**One-way travel time to work**	
Less than 15 minutes	304 (40.3%)
15- 30 minutes	273 (36.2%)
More than 30 minutes	161 (21.3%)
Missing	17 (2.2%)

Nurses were the largest professional group surveyed at PHC centers (32.7%), followed by generalists (23.0%) and medical specialists (21.7%). More than a third of surveyed providers (35.9%) had been working in the PHC setting for one to five years. Over half of the respondents (56.7%) were working full-time at the PHC centers and an additional third were working on a part-time basis. Two fifth of them (40.3%) reported less than 15 minutes one-way travel time to work.

### Likelihood to quit

Likelihood to quit within the next 1–3 years was investigated among providers at PHC centers; close to two fifth of the respondents (39.4%) indicated likelihood to quit their jobs (likely or very likely) and an additional 13.4% were not sure about staying or quitting (Table 2).

**Table 2:** Providers’ reported likelihood to quit and burnout level

**Variables**	**Frequency (N)**	**Percentage (%)**
**Likelihood to quit**		
Likely	285	39.4
Not sure	97	13.4
Unlikely	342	47.2
**Burnout Level**		
**Emotional Exhaustion (EE)**		
High	165	23.2
Medium	126	17.7
Low	421	59.1
**Depersonalization (DP)**		
High	98	13.8
Medium	110	15.5
Low	502	70.7
**Personal Accomplishment (PA)**		
High	132	18.7
Medium	116	16.4
Low	459	64.9

Surveyed providers gave a variety of reasons behind willingness to quit their job, the top five of which were: poor salary (54.4%), better job opportunities outside the country (35.1%), lack of professional development (33.7%), job instability (31.6%) and the lack of support from the administration (31.2%). Moreover, when asked about the reasons behind their choice to work in the current PHC center, the following reasons were the most commonly cited by respondents: the nature of the job, potential benefits to the family, proximity to household and area of work.

Also displayed in Table 2 are the findings related to the three subscales of burnout. Analysis reveals that experience of high burnout among providers is related to EE in almost a quarter of the respondents, DP in 13.8% of respondents, and PA in 18.7% of the respondents.

### Bivariate analyses with likelihood to quit

Table 3 reports bivariate associations between characteristics of providers and likelihood to quit. Higher burnout levels on all three subscales [EE (p-value<0.001), PA (p-value=0.010), and DP (p-value=0.002)] were significantly associated with likelihood to quit.

**Table 3:** Association between likelihood to quit and providers’ characteristics (N=755)

			**Likelihood to quit**						**P-value**
			**Likely**		**Not sure**		**Unlikely**		
			**N**	**%**	**N**	**%**	**N**	**%**	
**Burnout**	**EE**	Low	136	32.5%	58	13.9%	224	53.6%	<0.001
		Moderate	50	40.3%	19	15.3%	55	44.4%	
		High	94	57.0%	18	10.9%	53	32.1%	
	**PA**	Low	159	34.9%	62	13.6%	234	51.4%	0.010
		Moderate	57	49.1%	16	13.8%	43	37.1%	
		High	63	47.7%	16	12.1%	53	40.2%	
	**DP**	Low	175	35.2%	72	14.5%	250	50.3%	0.002
		Moderate	50	45.5%	13	11.8%	47	42.7%	
		High	55	56.1%	9	9.2%	34	34.7%	
**Training/Education**	**Education**	High school or lower /vocational/ diploma	45	42.1%	13	12.1%	49	45.8%	0.045
		Nursing degree	71	34.3%	24	11.6%	112	54.1%	
		University degree	55	52.4%	16	15.2%	34	32.4%	
		Medical degree	44	35.5%	21	16.9%	59	47.6%	
		Medical Specialty	68	38.2%	23	12.9%	87	48.9%	
	**Number of years since graduation**	10 years or less	142	44.8%	55	17.4%	120	37.9%	<0.001
		11-20 years	75	40.5%	22	11.9%	88	47.6%	
		21-30 years	48	32.4%	14	9.5%	86	58.1%	
		More than 30 years	12	21.4%	5	8.9%	39	69.6%	
	**Number of years working in primary care centers**	Six months to 1 year	41	45.1%	16	17.6%	34	37.4%	0.003
		1-5 years	113	43.1%	39	14.9%	110	42.0%	
		6-10 years	69	41.6%	21	12.7%	76	45.8%	
		More than 10 years	61	29.9%	21	10.3%	122	59.8%	
**Socio-economic variables**	**Age**	18-25	40	48.8%	15	18.3%	27	32.9%	0.001
		26-35	97	44.3%	32	14.6%	90	41.1%	
		36-45	86	43.2%	24	12.1%	89	44.7%	
		46-55	49	27.7%	20	11.3%	108	61.0%	
		>55	13	31.0%	4	9.5%	25	59.5%	
	**Marital status**	Single	98	45.6%	40	18.6%	77	35.8%	0.001
		Married	174	36.9%	51	10.8%	247	52.3%	
		Other	11	31.4%	6	17.1%	18	51.4%	
	**Children**	No	135	44.6%	54	17.8%	114	37.6%	<0.001
		Yes	150	35.6%	43	10.2%	228	54.2%	
**Work-related variables**	**Current position in PHC**	Generalist	59	36.4%	25	15.4%	78	48.1%	0.025
		Medical specialist	61	39.6%	22	14.3%	71	46.1%	
		Nurse	88	36.2%	29	11.9%	126	51.9%	
		Allied health professionals	51	45.9%	8	7.2%	52	46.8%	
		Other professional	26	48.1%	13	24.1%	15	27.8%	
	**Number of years working in this PHC**	Six months to 1 year	58	43.6%	23	17.3%	52	39.1%	0.003
		1-5 years	136	42.1%	48	14.9%	139	43.0%	
		6-10 years	52	37.1%	14	10.0%	74	52.9%	
		More than 10 years	33	28.7%	10	8.7%	72	62.6%	
	**Number of work hours per week**	1-10 hours	71	41.5%	31	18.1%	69	40.4%	0.013
		11-30 hours	30	30.6%	17	17.3%	51	52.0%	
		More than 30 hours	85	38.8%	19	8.7%	115	52.5%	
**Living arrangements**	**Travel time**	Less than 15 minutes	97	32.3%	40	13.3%	163	54.3%	0.015
		15-30 minutes	119	44.7%	37	13.9%	110	41.4%	
		More than 30 minutes	69	43.9%	20	12.7%	68	43.3%	
	**Feel as part of the community**	Yes	235	37.4%	87	13.8%	307	48.8%	0.023
		No	21	60.0%	2	5.7%	12	34.3%	
		Not sure	29	50.9%	8	14.0%	20	35.1%	

Likelihood to quit differed by level of education (p-value=0.045) and number of years since graduation (p-value <0.001). The pattern of correlation was such that providers with more specialized education and more years of work experience were less likely to quit. The number of years working in PHC was also shown to have a significant association with likelihood to quit (p-value=0.003), with 10 years of experience being the cutoff point; providers who had less than 10 years were more likely to quit than those with more years of experience. Among demographic variables, analysis reveals a significant (p-value=0.001) U-shaped relationship between age and likelihood to quit, whereby the younger (18–25 years) and older (more than 55 years) were more likely to quit than other age groups. Both marital status (p-value=0.001) and having children (p-value<0.001) were correlated with likelihood to quit, with married providers and those with children being less likely to quit.

In terms of work position, likelihood to quit was highest among less specialized providers, including allied health professionals (technicians and assistants), as well as “other health professionals”, with proportions of 45.9% and 48.1%, respectively. Nurses expressed the lowest likelihood to quit their job (36.2%, p-value=0.025). Moreover, likelihood to quit was significantly associated with the number of years spent in the current PHC center of employment (p-value=0.003), with providers having worked ‘six months to one year’ being more likely to quit that those who worked for more years.

Sense of belonging (p-value = 0.023) showed to be a significant predictor of likelihood to quit among PHC providers. Moreover, one-way travel time from place of residence to the PHC center was also significantly associated with likelihood to quit (p-value = 0.015); the difference was especially noted at 15 minutes, whereby those who traveled less than 15 minutes were less likely to quit than the rest.

### Multinomial logistic regression

A multinomial logistic regression was conducted to examine the factors significantly associated with likelihood to quit among the PHC providers (Table 4). The goodness-of-fit statistics (Pearson and Deviance) were not significant, suggesting that the data fits the presented model. Two sub-scales of burnout were significantly associated with likelihood to quit: EE and PA. Those with high level of burnout on the EE subscale had 3.46 times the odds of quitting compared to respondents with low level of EE (95% CI = 2.00-5.99; p-value<0.001). Those with a moderate level of burnout on the EE subscale had 1.72 times the odds of quitting compared to those with low level of burnout (95% CI = 0.93-3.15; p-value 0.082).

**Table 4:** Regression model testing for predictors of likelihood to quit

	Likelihood to Quit**							
	**Likely vs. Not likely**				**Not sure vs. Not likely**			
	**OR**	**CI**		**P-value**	**OR**	**CI**		P-value*
		**Lower**	**Upper**			**Lower**	**Upper**	
**EE level**								
High vs. low	3.46	2.00	5.99	**<0.001**	0.78	0.31	1.96	0.596
Moderate vs. low	1.72	0.93	3.15	0.082	1.00	0.42	2.35	0.997
**PA level**								
High vs. low	3.05	1.67	5.56	**<0.001**	2.42	1.06	5.48	**0.035**
Moderate vs. low	1.78	0.98	3.23	0.060	0.97	0.40	2.36	0.944
**Education**								
Intermediate/high school/vocational or diploma vs. Medical specialty	1.63	0.71	3.78	0.253	2.27	0.74	6.98	0.153
Nursing degree vs. Medical specialty	0.84	0.36	1.94	0.681	1.03	0.31	3.47	0.962
University degree vs. Medical specialty	2.48	1.02	6.08	**0.046**	2.34	0.70	7.82	0.169
Medical degree vs. Medical specialty	1.12	0.61	2.06	0.712	1.89	0.88	4.05	0.101
**Number of work hours per week**								
1 to 10 hours vs. 31 and up hours	1.83	0.92	3.64	0.083	3.15	1.22	8.15	**0.018**
11 to 30 hours vs. 31 and up hours	0.82	0.42	1.62	0.568	2.00	0.81	4.94	0.135
**Number of years working in PHC**								
Six months to 1 year vs. More than 10 years	3.88	1.36	11.10	**0.011**	3.13	0.82	11.94	0.095
1 to 5 years vs. More than 10 years	1.60	0.81	3.15	0.179	1.44	0.58	3.60	0.437
6 to 10 years vs. More than 10 years	1.99	1.03	3.84	**0.041**	1.21	0.47	3.09	0.690

*P-values below 0.05 are significant and therefore in bold font.

** Goodness-of-fit: Pearson’s (chi-square = 7325.0, df= 676, p-value = 0.057) and Deviance (chi-square = 645.8, df=676, p-value = 0.793).

Respondents with high level of burnout on the PA subscale had 3.05 times the odds of quitting (95% CI = 1.67-5.56; p-value <0.001) and 2.42 times the odds of being unsure whether to stay or quit (95% CI = 1.06-5.48; p-value 0.035) as compared to respondents with low level PA. With regards to education, respondents who had a university degree had 2.48 times the odds of quitting compared to those with a medical specialty (95% CI = 1.02-6.08; p-value 0.046).

Regarding the number of work hours per week, those who worked 1 to 10 hours had 3.15 times the odds of being unsure of their likelihood to quit as compared to those who worked more than 30 hours (95% CI = 1.22-8.15; p-value 0.018). A couple of findings did not reach the level of significance but are worth noting: Increases in the number of work hours per week was associated with reduced likelihood to quit and full-time employment status served as a protective factor in terms of likelihood to quit.

As for number of years working in PHC, providers who had worked ‘Six months to one year’ had 3.88 times the odds of quitting as compared to those who had worked more than 10 years (95% CI = 1.36-11.10; p-value 0.011). Moreover, those who had worked 6 to 10 years had 1.99 times the odds of being likely to quit as compared to those who had worked more than 10 years at PHC centers (95% CI = 1.03-3.84; p-value 0.041).

## Discussion

This study is the first of its kind to systematically investigate factors significantly affecting health providers’ retention at PHCs in Lebanon and one of the rare regional attempts to examine this important issue. Results suggest that two of every five PHC providers expressed likelihood to quit their job and an additional 13.4% are undetermined whether to stay or leave. Such a finding is problematic considering the existing PHC HHR retention crisis observed worldwide and the ongoing attrition of health providers from low and middle income countries to high income countries [11,33-35]; this is of specific relevance to Lebanon as well considering the migration trend of HHR to Gulf countries [36]. Furthermore, it is indicative of a volatile workforce and flags a policy priority to investigate and act upon the underlying causes behind health providers’ expressed likelihood to quit their jobs.

One in four PHC providers suffers from a high level of burnout related to EE and one in seven suffers from a high level of burnout related to DP. The observed level of burnout among PHC providers is worrisome for two reasons. First, PHC settings are anticipated to place a lower degree of stress on HHR as compared to the high stress environment of acute hospital care, in which patients are of higher acuity level [37]; however, our findings indicate a relatively elevated degree of stress. Second, there exists a significant and strong association between professional burnout and likelihood to quit. Literature extensively establishes the mediating role of burnout when investigating healthcare providers’ turnover within the hospital and PHC setting, as the level of burnout presents as a predictor of job satisfaction [12,29,38].

The findings do not only reflect the presence of elements in the work culture that are precipitating burnout on PHC providers, but also signal a priority issue for PHC managers to support their staff in order to mitigate the effects of this burnout. Strategies that involve securing adequate staffing, appropriate infrastructure, attractive benefits, supportive work environments, along with professional human resource development practices have shown to allow health workers to respond more effectively to the demands of their jobs, and thus enhance their retention [34,39,40]**.** Retention strategies must, however, be adapted to meet the needs of HHR working in different settings; a ‘one-coat-fits-all’ approach is doubtful to succeed [39]. Considering the top reasons for quitting that were reported in this study, there should be improvements directed towards remuneration policies. It is recommended that flexibility be built into the current remuneration system at PHC centers, where practices such as but not limited to employment of salary ranges may attract health providers to understaffed and/or underserved areas. More importantly, however, is the pressing need for increased attention towards enhancing education and professional development and boosting the sense of job stability and security amongst providers, as a means for improving role satisfaction within the PHC centers. This recommendation is consistent with literature which has highlighted the comparable importance of non-financial incentives to financial incentives in influencing health workers’ decisions to remain in the job [41].

Furthermore, stakeholders within the PHC setting are also invited to reflect on the observed U-shaped pattern between age and likelihood to quit. While it is anticipated to observe a higher likelihood to quit amongst older providers aged more than 55 years as they are approaching the age of retirement, it is alarming to observe a higher likelihood of quitting amongst younger providers aged 18–25 years. Although this age group includes new graduates and young professionals who tend to have a high degree of mobility [42], they are the ones with greatest potential to establish, through appropriate support and mentorship, lifelong careers in PHC. Efforts to provide safe and conducive work environments that facilitate the integration of new recruits may be wielded through support programs that ease transition into the new work culture. Developed models to attract and retain newly qualified nurses in primary care entail provision of support during the first months of employment, through preceptors and clinical induction programs [14,43,44]. Such initiatives have proven their effectiveness in the hospital sector, for example, where they were associated with improvements in self-confidence in patient care provision, positive results in new nurses’ competency and more importantly reduced turnover and increased retention rates [45,46].

The inversely proportional relationship between likelihood to quit amongst health providers and job tenure – with the shortest tenure (six months to one year) having the highest likelihood to quit – further supports the need for new staff mentorship and support programs. This is pivotal since almost half of the respondents have been working for a relatively short period of time in the current PHC center of employment and are therefore prone to display a higher likelihood of quitting. This relationship with institutional tenure could be attributed to providers’ dissatisfaction with the nature of the job as it compares with their expectations during the early employment period. A study conducted by Stewart et al., showed that the number of years nurses were employed to be significantly related to likelihood to quit; RNs who had been employed less than 2 years in their current positions were 3 times more likely to intend to quit than RNs employed 20 years or more [47]. In another study conducted on nurse migration in Lebanon, El-Jardali et al. found that one out of five registered nurses migrates out of Lebanon within 1–2 years of graduation [36]. On the other hand, the observed decreased likelihood to quit with increasing tenure may be indicative of a sense of organizational commitment amongst those providers- a finding that is supported in literature [17,48].

Another important consideration relates to the educational programs of health providers which may be more focused on preparing them for services in the hospital sector and are potentially making them less acquainted to serving in community settings [49-51]. Moreover, nursing students tend to favor the hospital setting due to a self-perceived lack of clinical experience that drives them to seek support more readily available in a hospital workplace [52]. Studies have shown that changes made to the medical school and residency programs, to better incorporate skills relevant to the PHC setting, have resulted in an increase in the number of providers hired at PHC centers [53]. This underscores the critical role that orientation, mentorship and support programs play in supporting PHC providers, especially the younger new graduates of them.

Likelihood to quit is significantly associated with position within the PHC center as the more skilled groups of providers were less likely to quit compared to allied health professionals. A key explanation relates to the fact that allied health professionals receive a relatively lower salary compared to other skilled professionals. Another possibility may revolve around increased job satisfaction stemming from direct contact with patients [54]. Skilled professionals, including physicians and nurses, due to their contact with patients, may acquire an intrinsic sense of reward that might mitigate their likelihoods to quit. This finding exposes a need for managers of PHC centers to dedicate targeted programs to professional with transferrable skills, so as to successfully retain them within their institutions.

Geographical location and sense of belonging to the community are shown to be two significant factors associated with likelihood to quit. More specifically, travel time significantly determined the likelihood of PHC providers to quit, with 15 minutes of transportation being the cutoff point between being likely and unlikely to quit. This could be explained by the poor public transportation conditions, high cost of gas, and lack of transportation allowances on behalf of the PHC centers in Lebanon. Numerous studies have pointed to geographic location as an important indicator of intention to leave a position within PHC centers and hospitals alike, as it is attributed to a desire for less commute time, as well as community belonging [15,47,51,53]. These results are suggestive of a need to staff PHC centers with providers residing in nearby, which may be particularly useful for PHC centers in remote rural areas of Lebanon; employees may be more easily retained with enhanced sense of belonging and easy commute.

This study has a number of shortcomings that warrant mentioning. First, despite the best efforts of the research team to pilot test the survey questionnaire, it cannot be ascertained that all providers were able to understand all the questions; particularly the less educated providers. Second, the study achieved an overall response rate of 60%. Although this response rate falls within the acceptable range for similar studies, the research team is not able to confirm whether the survey respondents are any different than non-respondents. It has to be noted though that most of the non-respondents are those that work on a part-time or casual basis at surveyed PHC centers. Third, despite the presence of some similarities between the private and the public PHC services in Lebanon, the findings of the study are only generalizable to the private PHC sector. Fourth, as a cross-sectional study, it does not allow for examining a causal relationship between the dependent variable of interest, likelihood to quit, and the factors that might affect its presence.

## Conclusions

This study has identified key findings related to the retention of human health resources in primary healthcare centers in Lebanon, a topic that has not been investigated much in the region. Several recommendations can be made to managers at PHC centers with the aim of stabilizing the workforce and ensuring its longevity. Retention strategies should be developed, implemented and evaluated at various centers and for all provider groups, but more importantly for those who appear to be at high risk of quitting. Targeted mentorship and support programs should be dedicated to new graduates and younger recruits who are particularly prone to quitting their jobs.

PHC centers must invest in enhancing the quality of their work environment to reduce stressors in the workplace and improve HHR satisfaction. Globalization and regional competition necessitate the enhancement of HHR compensation and access to professional development opportunities.

The renewed global commitment to PHC as the linchpin of individual and population health does not only necessitate immediate action on the findings of this study but also requires the intervention of multiple stakeholders, including: managers, syndicates and orders, the Ministries of Population Health and Social Affairs, educational and research institutions. The study also paves the way for future studies both locally and regionally. While some of the findings and recommendations may be context specific, others certainly would apply to other countries in the region.

Access to people-centered primary healthcare is widely recognized as an important facilitator of overall population health, it is of pivotal importance that PHC stakeholders exercise all due diligence to foster a work milieu that is conducive to staff retention and would allow for sustainability in provision of health services.

## Competing interests

None to declare for any of the authors.

## Authors’ contributions

MA: Conceptualized the study, wrote the proposal, secured funding, lead all aspects of data collection and analysis and prepared the first draft of the manuscript. He approved the final submitted version of this manuscript. SS: Conceptualized the study, contributed to data analysis of collected information and helped in the write up of the manuscript. He approved the final submitted version of this manuscript. FE: Helped with the conceptualization the study, contributed to the write up of the funded grant and significantly contributed to analysis and write up of the manuscript. He reviewed the manuscript and approved the final submitted version. HD: Lead the statistical analysis carried out in this study and contributed to the write up of the manuscript. He reviewed the manuscript and approved the final submitted version. YM: Contributed to data entry and analysis. Provided valuable support with the referencing and editing of the final version of the manuscript. All authors read and approved the final manuscript.

## Pre-publication history

The pre-publication history for this paper can be accessed here:

http://www.biomedcentral.com/1472-6963/12/419/prepub
